# Feedback Signals in Myelodysplastic Syndromes: Increased Self-Renewal of the Malignant Clone Suppresses Normal Hematopoiesis

**DOI:** 10.1371/journal.pcbi.1003599

**Published:** 2014-04-24

**Authors:** Thomas Walenda, Thomas Stiehl, Hanna Braun, Julia Fröbel, Anthony D. Ho, Thomas Schroeder, Tamme W. Goecke, Björn Rath, Ulrich Germing, Anna Marciniak-Czochra, Wolfgang Wagner

**Affiliations:** 1 Helmholtz Institute for Biomedical Engineering, RWTH Aachen University Medical School, Aachen, Germany; 2 Interdisciplinary Center of Scientific Computing (IWR), Institute of Applied Mathematics, University of Heidelberg, Heidelberg, Germany; 3 Department of Hematology, Oncology and Clinical Immunology, Heinrich-Heine-University Düsseldorf, Düsseldorf, Germany; 4 Department of Medicine V, Medical Center, University of Heidelberg, Heidelberg, Germany; 5 Department of Obstetrics and Gynecology, RWTH Aachen University Medical School, Aachen, Germany; 6 Department for Orthopedics, RWTH Aachen University Medical School, Aachen, Germany; University of California, Irvine, United States of America

## Abstract

Myelodysplastic syndromes (MDS) are triggered by an aberrant hematopoietic stem cell (HSC). It is, however, unclear how this clone interferes with physiologic blood formation. In this study, we followed the hypothesis that the MDS clone impinges on feedback signals for self-renewal and differentiation and thereby suppresses normal hematopoiesis. Based on the theory that the MDS clone affects feedback signals for self-renewal and differentiation and hence suppresses normal hematopoiesis, we have developed a mathematical model to simulate different modifications in MDS-initiating cells and systemic feedback signals during disease development. These simulations revealed that the disease initiating cells must have higher self-renewal rates than normal HSCs to outcompete normal hematopoiesis. We assumed that self-renewal is the default pathway of stem and progenitor cells which is down-regulated by an increasing number of primitive cells in the bone marrow niche – including the premature MDS cells. Furthermore, the proliferative signal is up-regulated by cytopenia. Overall, our model is compatible with clinically observed MDS development, even though a single mutation scenario is unlikely for real disease progression which is usually associated with complex clonal hierarchy. For experimental validation of systemic feedback signals, we analyzed the impact of MDS patient derived serum on hematopoietic progenitor cells *in vitro*: in fact, MDS serum slightly increased proliferation, whereas maintenance of primitive phenotype was reduced. However, MDS serum did not significantly affect colony forming unit (CFU) frequencies indicating that regulation of self-renewal may involve local signals from the niche. Taken together, we suggest that initial mutations in MDS particularly favor aberrant high self-renewal rates. Accumulation of primitive MDS cells in the bone marrow then interferes with feedback signals for normal hematopoiesis – which then results in cytopenia.

## Author Summary

Myelodysplastic syndromes are diseases which are characterized by ineffective blood formation. There is accumulating evidence that they are caused by an aberrant hematopoietic stem cell. However, it is yet unclear how this malignant clone suppresses normal hematopoiesis. To this end, we generated mathematical models under the assumption that feedback signals regulate self-renewal and proliferation of normal and diseased stem cells. The simulations demonstrate that the malignant cells must have particularly higher self-renewal rates than normal stem cells – rather than higher proliferation rates. On the other hand, down-regulation of self-renewal by the increasing number of malignant cells in the bone marrow niche can explain impairment of normal blood formation. In fact, we show that serum of patients with myelodysplastic syndrome, as compared to serum of healthy donors, stimulates proliferation and moderately impacts on maintenance of hematopoietic stem and progenitor cells *in vitro*. Thus, aberrant high self-renewal rates of the malignant clone seem to initiate disease development; suppression of normal blood formation is then caused by a rebound effect of feedback signals which down-regulate self-renewal of normal stem and progenitor cells as well.

## Introduction

Myelodysplastic syndromes are clonal disorders which are characterized by ineffective hematopoiesis, peripheral cytopenia and a high risk of disease progression towards acute myeloid leukemia (AML) [1]–[3]. They arise from an aberrant HSC that gains growth advantage over normal hematopoiesis resulting in clonal expansion [4], [5]. The pathogenesis of this disease is still unclear, and no curative treatment has been developed with the exception of stem cell transplantation [6]–[8]. So far, research has particularly focused on cell-intrinsic modification of MDS cells: mutations and molecular aberrations have been identified which seem to increase proliferation of the malignant clone [9], [10]. On the other hand, defects might also emerge as a result of an abnormal microenvironment [11]–[13]. Mesenchymal stromal cells (MSCs) show intrinsic growth deficiency in MDS [14] and fail to support hematopoiesis [13]. It has been suggested that MDS is also associated with increased apoptosis rates of normal bone marrow cells [3], [15]. So far, the mechanisms that suppress normal hematopoiesis remain unclear, as there is no evidence that the bone marrow niche is completely filled by the malignant clone [4], [16].

Self-renewal and differentiation of HSCs need to be tightly controlled according to the physiological needs [17]. For this purpose, feedback signals may either be derived from the immediate bone marrow microenvironment or by systemically released factors. The highest self-renewal rate is expected for the long-term repopulating HSCs (LT-HSCs) which predominantly remain dormant under steady-state conditions [18]. Yet, self-renewal and differentiation are also prerequisites of short-term repopulating stem cells (ST-HSCs), multipotent progenitor cells (MPPs), committed progenitor cells (CPCs) and precursors [19], [20]. In analogy, cells derived from the aberrant MDS clone may also display a hierarchy of self-renewal and differentiation: this is in line with the concept of cancer stem cells – or tumor initiating cells – which then reveal further differentiation and heterogeneity [21], [22]. It is generally anticipated that proliferation rates are higher in malignant cells. On the other hand, several mutations seem to affect the self-renewal in MDS [23], [24] – yet, this is difficult to study under *in vivo* conditions.

Mathematical modeling is a powerful tool to study interaction of different cell types and the impact of feedback signals [18], [25], [26]. Based on the biological context several models have been proposed to study the impact of feedback signals on system stability and regenerative properties. Theoretical and experimental studies on the olfactory epithelium [27], [28] as well as theoretical considerations of self-renewing cell lineages [29] demonstrate the necessity of feedback signals for system stability and efficient regeneration. We have recently proposed mathematical models describing activation of the HSC-pool upon hematopoietic stem cell transplantation (HSCT). These models indicated that feedback signals for self-renewal and proliferation are important. In particular, the increased self-renewal rates of immature cells facilitate efficient hematopoietic reconstitution [18], [30]. Similar results have been obtained for the olfactory epithelium [27]. Subsequently, we have shown that patient serum obtained during aplasia after HSCT has impact on hematopoietic progenitor cells (HPCs) *in vitro*: it significantly increased proliferation, maintenance of the primitive immunophenotype and expansion of colony forming units (CFUs) [31]. These findings supported the notion that systemically released factors contribute to regulation of stem cell function.

In the current work, we conceived a mathematical model to simulate development of MDS with particular focus on self-renewal and proliferation of the aberrant clone. MDS is a very heterogeneous disease. Furthermore, multiple mutations contribute to a complex clonal hierarchy during disease progression and many parameters are so far not well defined in specific cellular subpopulations. In this regard, we aimed for a conceptional approximation how the malignant clone interferes with normal hematopoiesis – irrespective of specific MDS subtypes or hematopoietic cell lineages as well as multiple mutation scenarios. Existence of the proposed feedback signals was then substantiated using serum of MDS patients.

## Methods

### Ethics statement

The use of all human materials was performed after written consent and according to the guidelines approved by the local Ethic Committees: CD34^+^ cells were isolated from umbilical cord blood (CB; Permit Number: EK187/08; RWTH Aachen University); CD34^+^ cells and MSCs were also isolated from bone marrow (BM) during surgical intervention (Permit Numbers: EK300/13 and EK128/09; RWTH Aachen University); and serum from MDS patients or healthy controls was collected in Düsseldorf and Aachen, respectively (Permit numbers: 2972 and EK206/09).

### Mathematical modeling

**Figure 1:**
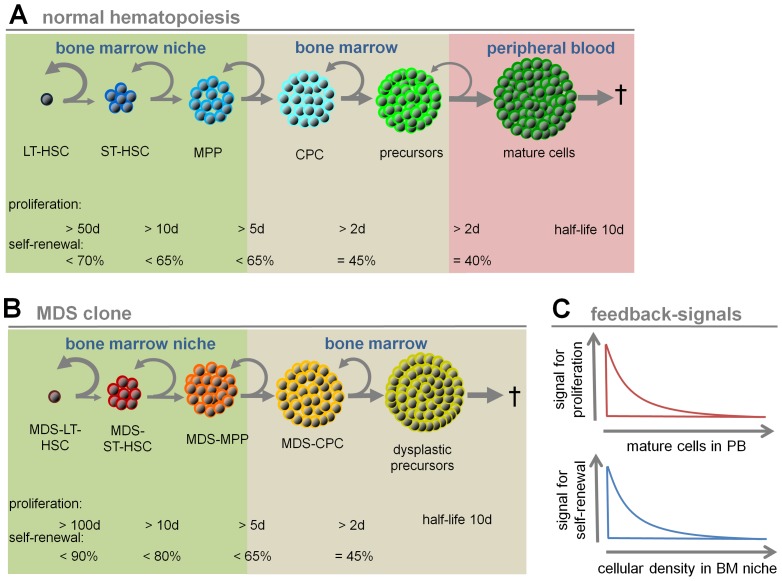
Schematic presentation of parameters of the mathematical model. (A) Normal hematopoiesis is assumed in six maturation steps: long-term repopulating stem cells (LT-HSCs), short-term repopulating stem cells (ST-HSCs), multipotent progenitor cells (MPPs), committed progenitor cells (CPCs), precursors and mature cells. Grey arrows exemplarily demonstrate the balance of self-renewal and differentiation at each step. The maximal proliferation rates and the maximal self-renewal rates were adopted from our previous work [18]. (B) MDS development is simulated in five compartments (MDS-LT-HSCs, MDS-ST-HSCs, MDS-MPPs, MDS-CPCs and dysplastic precursors). For MDS-LT-HSCs, we assume that the proliferation rate is even lower than for normal LT-HSCs (maximal once per 100 d *versus* maximal once per 50 d), whereas the self-renewal rate is higher (up to 90% *versus* up to 70%). (C) Signal intensity for proliferation is assumed to be inversely correlated to mature cells in peripheral blood, whereas the self-renewal signal is inversely correlated to the number of stem and progenitor cells in the BM-niche compartment. Both signals affect normal hematopoiesis as well as the malignant clone.

The mathematical model developed in this study considers interaction of normal hematopoiesis and myelodysplastic cells in the bone marrow. It is based on a previously proposed model of the hematopoietic system [18] that was extended to describe dynamics of aberrant clones, as in MDS development [26]. The model is based on a system of ordinary differential equations describing the flux of cells through different maturation stages for both normal and malignant cells. The structure of the model is depicted in Figure 1 and a detailed description of the model is given in Text S1.

### Isolation of hematopoietic progenitor cells

CD34^+^ cells were isolated from fresh umbilical cord blood using the human CD34 Micro Bead Kit (Miltenyi Biotec GmbH, Bergisch-Gladbach, Germany) as described before [31]. Alternatively, CD34^+^ cells were isolated from human bone marrow aspirate from the femur obtained during orthopaedic surgery.

### Isolation of mesenchymal stromal cells

MSCs were isolated from the *caput femoris* and cultured as described before [32], [33]. For co-culture experiments, we have used MSCs of passage 3 to 6 (10–15 population doublings).

### Serum samples from patients with myelodysplastic syndromes

Serum samples from 57 MDS patients and 5 healthy controls were obtained from the Department of Hematology of Heinrich Heine University in Düsseldorf. Additionally, serum of 12 healthy controls was obtained from the Department of Gynaecology at RWTH Aachen University. Generation of serum was performed as described in detail before [31]. Relevant patient data are summarized in Table 1 in Text S1.

### Culture conditions for HPCs

Hematopoietic progenitor cells were expanded for up to seven days as described previously [34] in StemSpan culture medium supplemented with 10 ng/mL stem cell factor (SCF; PeproTech GmbH, Hamburg, Germany), 20 ng/mL thrombopoietin (TPO; PeproTech), 10 ng/mL fibroblast growth factor 1 (FGF-1; PeproTech) and 10 µg/mL heparin (Roche GmbH, Mannheim, Germany) [32]. For co-culture experiments, addition of cytokines was not performed as MSCs alone activate proliferation. Culture medium was always supplemented with 10% serum of individual MDS patients or control samples as described in our previous work [31].

### Analysis of cell division history and immunophenotype

Freshly isolated CD34^+^ cells (either from CB or BM) were labelled with carboxyfluorescein diacetate N-succinimidyl ester (CFSE; Sigma-Aldrich, Hamburg, Germany) to monitor cell divisions as previously described [34]. After five days, CFSE intensity was measured by flow cytometry. For immunophenotypic analysis, cells were stained with CD34-allophycocyanin, CD133-phycoerythrin and CD45-V500 and analyzed using a FACS Canto II (BD) [32]. Further details on immunophenotypic analysis are provided in Text S1.

### Colony forming unit assay

Colony forming unit (CFU) frequency was determined to estimate culture expansion on HPCs. In brief, 12,500 CD34^+^ cells were grown for seven days in StemSpan medium supplemented with SCF, TPO, FGF, heparin and 10% patient serum. The progeny was harvested and analyzed in the CFU-assay as described before [31].

### Cytokine ELISA

Concentrations of SCF, TPO and FGF in patient serum were determined with RayBio Human ELISA Kits (RayBiotec, Norcross, GA, USA) according to the manufacturer's instructions. Concentration of erythropoietin (EPO) was measured by the laboratory diagnostic center of RWTH Aachen University with a chemoluminescent-immunometric assay (IMMULITE 1000 EPO).

### Statistics

All results are expressed as mean ± standard deviation (SD) or ± standard error of the mean (SEM). To estimate the probability of differences, we have adopted the two-sided Student's T-test. Probability value of p<0.05 denoted statistical significance.

## Results

### Increased self-renewal is essential in MDS

We propose a mathematical model to address the relevance of self-renewal and proliferation rates for MDS development. The model describes interaction of 1) normal hematopoietic cells, which progress along long-term repopulating stem cells (LT-HSCs), short-term repopulating stem cells (ST-HSCs), multipotent progenitor cells (MPPs), committed progenitor cells (CPCs), precursors and mature cells (Figure 1A), with 2) cells of the MDS clone which progress through analogous steps of differentiation except for mature cells (MDS-LT-HSCs, MDS-ST-HSCs, MDS-MPPs, MDS-CPCs and dysplastic precursors; Figure 1B). We assume that proliferation is regulated in normal and malignant cells by feedback signals acting on all developmental stages - it is inversely correlated with the number of mature cells in peripheral blood (PB). On the other hand, we assume that self-renewal is regulated by cellular density in a virtual stem cell niche occupied exclusively by the more primitive cells in the marrow – it is inversely correlated with the number of cells in the three more primitive compartments (LT-HSCs, ST-HSCs, MPPs, MDS-LT-HSCs, MDS-ST-HSCs, and MDS-MPPs; Figure 1C). A wide range of values of each parameter has been examined. The simulations consistently demonstrate that high self-renewal of MDS-initiating cells is crucial for MDS development. Only if MDS-LT-HSCs have a higher self-renewal potential than normal LT-HSCs, they eventually outcompete healthy hematopoiesis. In contrary, increased proliferation of MDS-cells alone is not sufficient. Notably, we have assumed that the proliferation rate of MDS-HSCs is lower than in normal LT-HSCs (maximal cell division rate every 100 versus every 50 days, respectively) - even then the MDS clone gains predominance if the self-renewal rate is higher than in normal LT-HSCs (maximal self-renewal rate 90% *versus* 70%, respectively). Nevertheless, high proliferation rates in MDS cells - although not required for establishment of the disease – would accelerate expansion of a cell population if self-renewal is also increased. Our results indicate that increased self-renewal is most essential for MDS, whereas an additional increase of proliferation accelerates the impairment of hematopoiesis.

### Evolution of MDS clone and suppression of normal hematopoiesis

**Figure 2:**
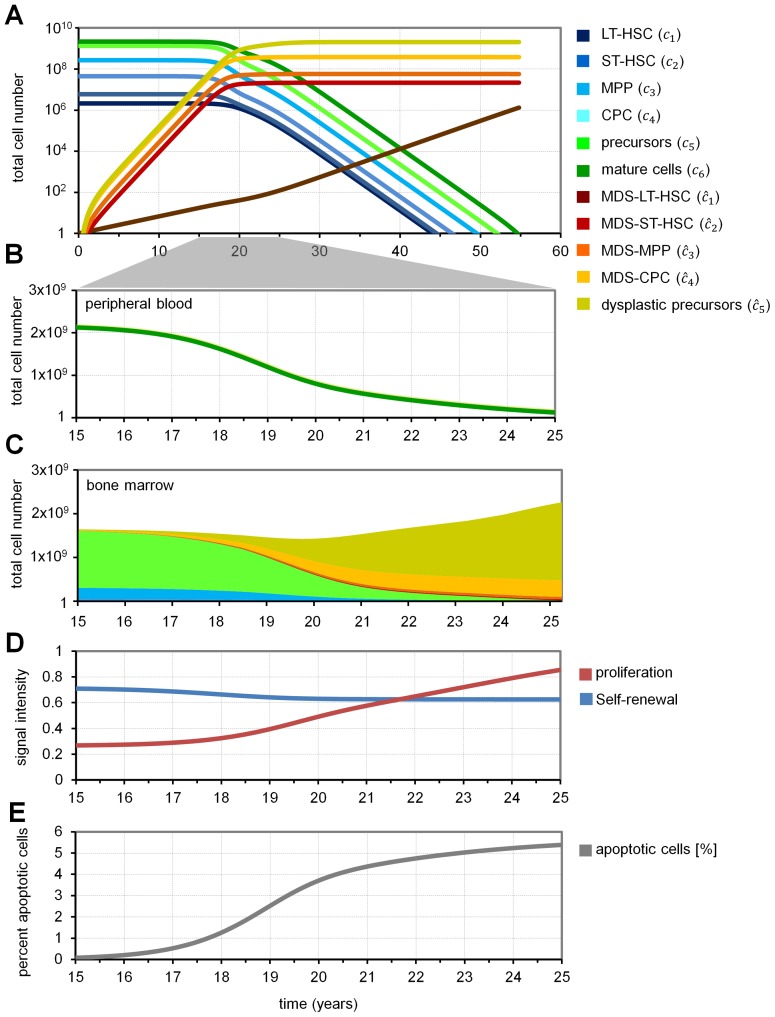
Simulation of MDS development. (A) Simulated MDS development under the assumptions depicted in Figure 1. Initially, a single MDS-LT-HSC is present. Evolution of cell numbers in the different compartments is simulated over 55 years. (B) A sharp decline of mature cells is achieved after about 17 years – this would correspond to clinical manifestation of MDS. (C) Simulated cellular composition in the BM at the relevant time frame. (D) Corresponding signal intensities for self-renewal and proliferation are presented. Self-renewal decays due to the accumulation of malignant cells in the BM-niche; proliferation is activated due to ineffective hematopoiesis. (E) The percentage of apoptotic cells in the bone marrow is presented.

MDS is usually a slow progressive disease which occurs particularly in elderly people. Simulated examples with input parameters derived from our previous work [18], [35] demonstrated that clonally derived MDS cells may increase over approximately 15 to 17 years without clinically relevant changes in bone marrow or blood counts (Figure 2A). After 17 years, the BM will contain about 1.66×10^−6^% MDS-LT-HSCs, 0.39% MDS-ST-HSCs, 1.12% MDS-MPPs, 5.38% MDS-CPCs and 8.15% dysplastic progenitors. Then, within few years, the number of mature cells in PB drops significantly (Figure 2B). Correspondingly, the percentage of normal hematopoietic cells in the bone marrow declines (Figure 2C). The simulated dynamics of disease development are as follows: 1) Initially, a single MDS cell expands very slowly due to higher self-renewal compared to normal LT-HSCs. 2) Consequently, the number of cells in the bone marrow niche increases which leads, via feedback signaling, to reduced self-renewal of cells in the niche. 3) This indirectly results in suppression of normal hematopoiesis and cytopenia. 4) The low number of mature cells triggers proliferation of normal and malignant cells and thereby enhances disease progression (Figure 2D). In this model, we consider apoptosis rates of mature cells and of dysplastic progenitors only. However, due to the increasing number of dysplastic precursors which die within 10 days, the percentage of apoptotic cells in the bone marrow increases to 5.6% (under the assumption that apoptosis takes 24 h; Figure 2E). Alternatively, we modeled MDS including further maturation of MDS cells and high apoptosis on the level of committed progenitors. We assume that MDS derived mature cells have higher apoptosis rates than normal mature cells (half-life time only 16 h), which is in line with higher apoptosis rates in bone marrow and peripheral blood observed in MDS patients [3], [15], [36]. These simulations lead to qualitatively similar results: in all cases, enhanced self-renewal of disease initiating cells is crucial for establishment of the disease. This indicates that increased apoptosis is compatible - but not required - for MDS development in our approach (Figure 1 in Text S1).

### CD34^+^ cells in MDS

The percentage of CD34^+^ cells in healthy bone marrow, low-risk MDS, and high-risk MDS was 1.4±0.2%, 3.4±0.7% and 7.8±1.9%, respectively (Figure 2 in Text S1). In our model, we assume that LT-HSCs, ST-HSCs, and MPPs, as well as MDS-LT-HSCs, MDS-ST-HSCs, and MDS-MPPs correspond to CD34^+^ cells – they are not pure stem cell fractions but they are all influenced by the self-renewal signal. The percentage of primitive cells is compatible with dynamics of the mathematical model, but it rapidly increases over time. It has been previously suggested that the percentage of blasts, defined as CD117^+^ or CD34^+^ cells, has prognostic value for survival [2], [37]. In this regard, it might be speculated that high-risk MDS is characterized by higher cell-intrinsic self-renewal.

### Serum from MDS patients stimulates proliferation of CD34^+^ cells

**Figure 3:**
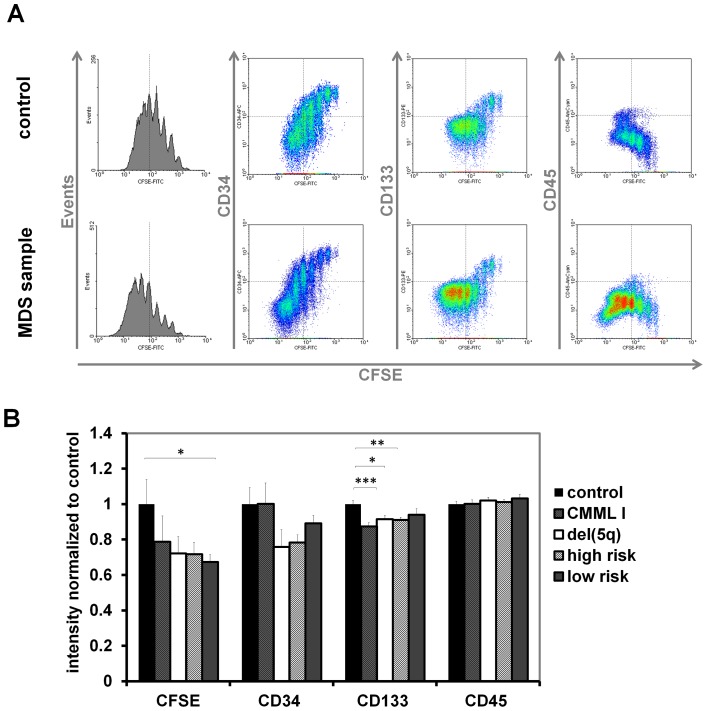
Serum of MDS patients stimulates proliferation of CD34^+^ cells. (A) CD34^+^ HPCs from umbilical cord blood were stained with CFSE and subsequently cultivated for five days *in vitro* in culture medium supplemented with 10% serum of individual patients or of healthy controls. Cell division history was monitored by residual CFSE-staining and dotted lines indicate five cell divisions. (B) Mean fluorescence intensities after five cell divisions were normalized to control samples. In each experiment, 57 MDS serum supplements and 17 control serum supplements were tested in parallel. Mean and standard deviation were calculated over measurements with three different cord blood samples (*p<0.05, **p<0.01, ***p<0.001).

**Figure 4:**
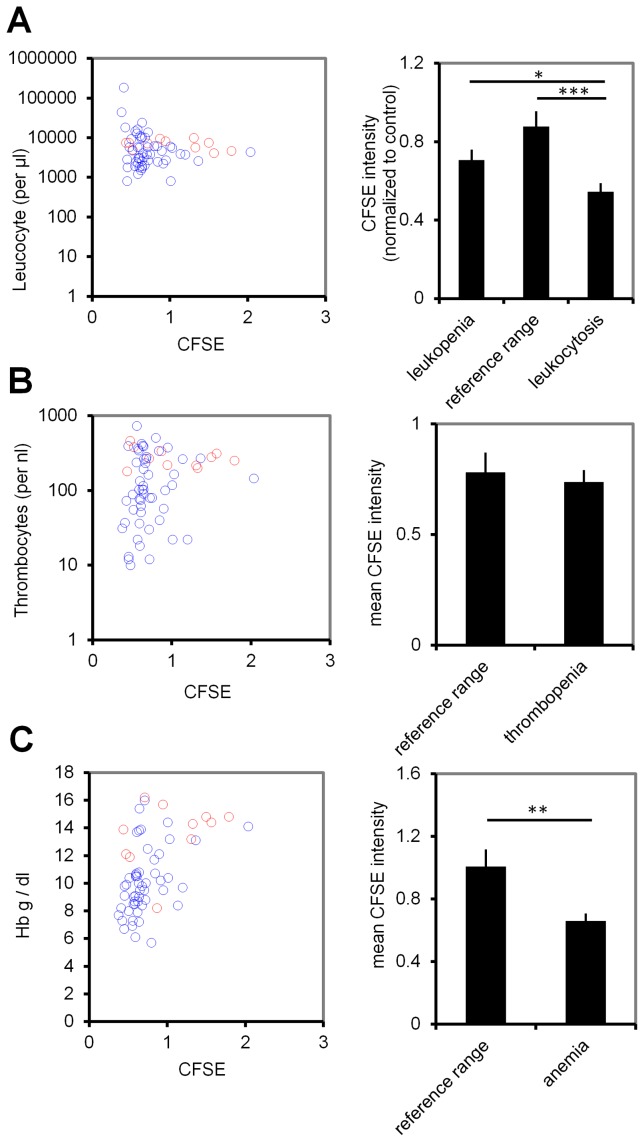
MDS serum of cytopenic patients enhances proliferation. The growth-stimulatory effect of MDS serum on CD34^+^ cord blood cells was correlated to (A) leucocyte counts, (B) thrombocyte counts, and (C) hemoglobin level (Hb). For statistical analysis of individual experiments (with different CB samples) CFSE intensity was normalized to healthy controls (blue circles resemble MDS serum, red circles individual control samples). Increased proliferation (i.e. less residual CFSE intensity) was observed with serum from leukopenic patients, leukocytotic patients (which rather resemble secondary AML with lower number of mature cells in PB), and anemic patients. Reference ranges used for classification: leucocytes: 3,800–10,500/µL; thrombocytes: 1.5×10^5^–4.5×10^5^/µL; Hb: male: 13–18 g/dL, female: 11–16 g/dL. Error bars represent SD (*p<0.05, **p<0.01, ***p<0.001).

Based on our mathematical model, we assumed that serum of MDS might comprise signaling molecules related to the systemic feedback which stimulate proliferation of CD34^+^ cells. These cells can be expanded *in vitro* – particularly if co-cultured with MSCs – but this is associated with further loss of stemness (Figure 3 in Text S1). We isolated serum of 57 MDS patients and 12 healthy controls. CB-derived CD34^+^ cells were then stained with CFSE and cultured in parallel with culture media supplemented with 10% of individual serum samples. After five days, the cells were analyzed by flow cytometry (Figure 3A). Overall, proliferation rate of CD34^+^ cells, and hence dilution of CFSE, was significantly higher in MDS serum (p = 0.007). When we subdivided MDS patients into high risk (sAML, RAEBI and RAEBII), low risk (RCMD and RCMD-RS), CMML I, and 5q chromosomal deletion increased proliferation was particularly observed using serum of low-risk MDS (p = 0.041; Figure 3B). These results were reproduced with all patient sera using HPCs of three different cord blood samples. Especially serum derived from leukopenic and anemic patients enhanced proliferation of HPCs (p = 0.05 and p = 0.004, respectively), whereas this trend was less pronounced with serum from thrombopenic patients (Figure 4). However, under co-culture conditions with MSCs, the growth-supporting effect of MDS serum was obscured by the overall growth-stimulation of stromal cells, even though we did not use cytokines in these experiments (Figure 4 in Text S1).

**Figure 5:**
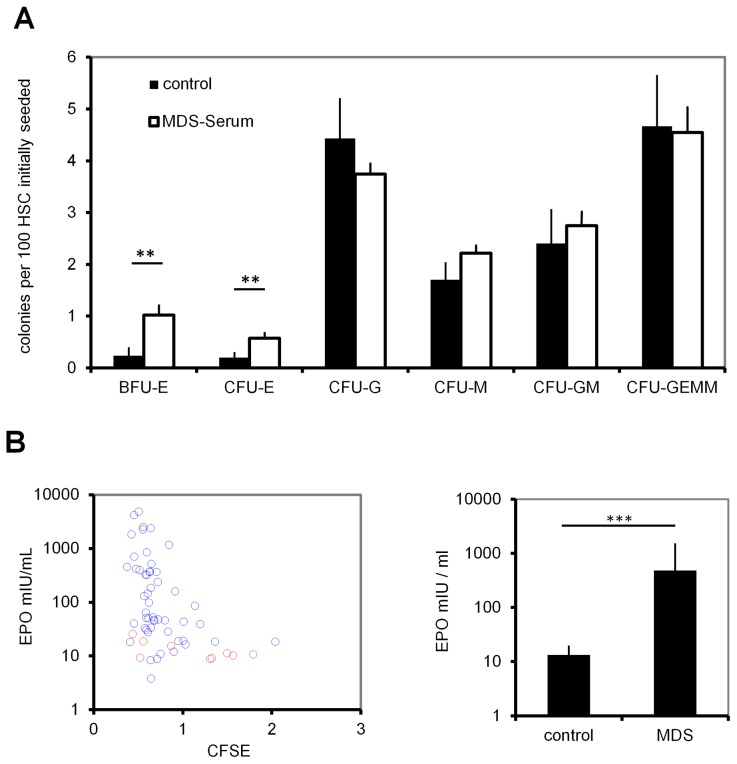
CFU-Frequency upon stimulation with MDS serum. (A) CD34^+^ HPCs from cord blood were cultured with 10% serum supplements of individual MDS patients or controls. After seven days, cells were re-seeded in methylcellulose medium and after two weeks, the numbers of erythrocyte (BFU-E and CFU-E), granulocyte (CFU-G), macrophage (CFU-M) and combined (CFU-GM and CFU-GEMM) colonies were counted. (B) Erythropoietin (EPO) concentration was significantly higher in MDS-derived serum samples. (C) The growth promoting effect on CD34^+^ cells was then plotted against the EPO concentration. CFSE intensity was normalized to healthy controls of the corresponding experiment. Error bars represent SD (*p<0.05, **p<0.01, ***p<0.001).

MDS is rather observed in elderly patients and it is conceivable that age-matched HPCs respond differently to feedback signals. Therefore, we performed two additional experiments with HPCs from adult bone marrow using all patient sera. In analogy to our results with CB-derived HPCs, BM-derived CD34^+^ cells revealed significantly higher proliferation if stimulated with serum from MDS-patients (del(5q): p = 0.0005; high-risk MDS: p = 0.0007; low-risk MDS: p = 0.0019; Figure 5 in Text S1). Overall, the results support the notion that the number of mature cells is inversely correlated with the proliferative effect of patient serum - which is in agreement with our model.

### Effects of MDS serum on immunophenotype and colony formation

Computer simulations demonstrated that our mathematical model recapitulates clinical observations under the assumption that the feedback signal for self-renewal decays if malignant cells accumulate in the stem cell niche. Therefore, we reasoned that MDS patient serum might also impair maintenance of the primitive immunophenotype *in vitro*. To this end, we have only measured expression of CD34 and CD133 in CB-HPCs which underwent five cell divisions to exclude bias by proliferation. In fact, CD34 and CD133 expression was moderately decreased with MDS serum (Figure 3B). In contrast, expression of CD45 was not influenced by MDS serum. A similar effect was also observed using BM-HPCs (Figure 5 in Text S1). Although effects of MDS serum on immunophenotype were rather moderate, they are in agreement with the proposed decrease of the self-renewal signal.

The impact of MDS serum was further analyzed with regard to maintenance of colony forming units (CFUs): CD34^+^ CB-HPCs were cultured *in vitro* for 7 days, and this was performed in parallel with medium supplemented with 10% of individual serum samples. The cells were then reseeded in methylcellulose medium and 12 to 14 days later, colony types and numbers were detected. Comparing MDS serum and control serum, no significant differences were found in colony-initiating cells (CFU-G, CFU-M, CFU-GM, and CFU-GEMM). Only the number of erythroid colonies (BFU-E and CFU-E) was significantly increased when exposed to serum of MDS patients (p = 0.004 and p = 0.02 respectively; Figure 5A). Thus, colony assays provide no support for the presence of circulating factors in MDS patient serum that increase colony formation initiated by the most primitive hematopoietic progenitors.

### Chemokines in MDS serum

The proposed feedback signals may involve growth factors. Therefore, we have analyzed serum levels of stem cell factor (SCF), thrombopoietin (TPO) and fibroblast growth factor (FGF) which support expansion of CD34^+^ cells *in vitro* [32], and of erythropoietin (EPO) which stimulates hematopoietic differentiation. Concentrations of SCF, TPO and FGF were higher in MDS serum than in control serum, but this trend did not reach statistical significance (Figure 6 in Text S1). However, the EPO-concentration was significantly higher in MDS patient serum and this is in line with previous reports (Figure 5B) [12], [38].

## Discussion

The results of this study support the notion that the balance of self-renewal and differentiation is skewed in MDS. This can be attributed to cell intrinsic modifications of the malignant clone. However, ineffective normal hematopoiesis indicates that the malignant clone also interferes with physiologic feedback loops - either mediated by the local environment or on systemic level [26], [39], [40]. Our mathematical model suggests that (1) cell-intrinsic increased self-renewal of the most primitive malignant cell clone is a prerequisite for disease development; (2) increased proliferation is less relevant and it may even be reduced in the malignant clone; and (3) suppression of hematopoiesis by reduced self-renewal due to accumulation of malignant cells is one possible mechanism to explain cytopenias in MDS.

Our mathematical model is based on differential equations to simulate the flux of the cells through different compartments [41], [42]. This approach is notoriously associated with simplifications: we have exemplarily chosen six compartments for normal hematopoiesis and five compartments for MDS cells. Experimental data do not facilitate precise distinction between particular stages. It is still under debate whether or not MDS is associated with a differentiation block and MDS cells are often able to generate differentiated, functional, albeit abnormal hematopoietic cells [1], [43]. Therefore, we have alternatively tested less or more compartments and these simulations resulted in qualitatively similar results. Our model does not distinguish between different hematopoietic lineages (such as lymphoid and myeloid differentiation). This is a simplification which assumes that all the lineages have similar proliferation and maturation structure and dynamics – most likely this does not represent physiological conditions, particularly with regard to the different MDS-subtypes, which may affect one or several lineages [43]. However, consideration of all hematopoietic lineages (or of specific MDS-subtypes) would entail very complex models which would not facilitate such clear conclusions. Another simplification is that we do consider modified apoptosis rates for mature cells and dysplastic progenitors only. It has been suggested that apoptosis rates are increased in MDS, especially in the more differentiated MDS cells in the bone marrow [3], [15]. Interestingly, this is also observed in our simulations due to cell death of dysplastic progenitors in the bone marrow. Alternatively, we assumed higher apoptosis rates in mature MDS cells and the simulations reveal similar cellular dynamics, too. The propensity to undergo premature programmed death does not appear to be the most important property, because if it were, there would be no clonal expansion at all [4], [44]. Model parameters were adapted based on our previous work [18], [35]. Such mathematical models cannot provide quantitative predictions for disease development. However, it is remarkable that despite the simplicity of our model, the time course of disease development is compatible with clinical observations. The long evolution over more than 17 years might explain why MDS occurs particularly in elderly patients.

Our model demonstrates that a malignant MDS clone can only outcompete normal hematopoiesis if the self-renewal rate of its primitive precursors is higher than in normal LT-HSCs. Higher proliferation of MDS initiating cells alone is not sufficient. This seems plausible since under steady state conditions the self-renewal of LT-HSCs equals 50% (i.e. upon division the daughter cells have equal probability to replenish the stem cell pool or to differentiate into ST-HSCs). If the self-renewal of MDS-ST-HSC was not higher than 50%, their population would not be able to expand. Therefore, we conclude that enhanced self-renewal is the most essential feature of MDS-LT-HSCs. Nevertheless, additionally increased proliferation rates of MDS cells, although not required for establishment of the disease, accelerates expansion of the dysplastic population.

Increased proliferation is often considered the most essential modification in malignant transformation [45], [46]. However, in our simulations the proliferation rate appears to be less relevant. Even under the assumption that MDS-LT-HSCs are less proliferative than in normal LT-HSCs, the normal hematopoiesis is eventually outcompeted. This might also explain the inefficiency of chemotherapeutic treatment in MDS as the conventional chemotherapy regimens preferentially eradicate proliferating cells. On the other hand, increased proliferation accelerates disease development and it is well documented that growth factors and hematopoietic stress recruit cells into cycle. Therefore, we have implemented a feedback signal for proliferation in our model. We assumed that this signal is triggered by the number of mature cells in the peripheral blood. In fact, MDS patient serum increased proliferation of CD34^+^ cells more than control serum and this was particularly observed in patients with severe aplasia and/or anemia, where the growth stimulatory effect, although moderate, was reproducible and significant. In analogy, we have recently described that serum from patients after autologous HSCT, which requires recruitment of the stem cell pool into proliferation for hematopoietic reconstitution, also stimulates proliferation of CD34^+^ cells [31]. These findings support the hypothesis that systemic feedback signals for hematopoietic proliferation are related to cell counts of mature cells.

Over the last decade, many cell intrinsic and extrinsic factors have been discussed for development of MDS [5], [11]. In fact, MDS often reveals mutations in genes which are involved in self-renewal of HSCs [47], [48]. Furthermore, epigenetic modifications – particularly DNA-methylation changes – seem to play a crucial role for disease development [49]–. We have demonstrated that epimutations in *DNMT3A*, a gene that is also frequently mutated in MDS, can mimic genomic mutations in AML [52]. We hypothesized that *DNMT3A* modifications represent early events - potentially even initiating events - that then entail less frequent mutations [52]. In fact, Shlush and coworkers have recently demonstrated that genomic mutations in *DNMT3A* arise early in AML evolution [53]. Somatic mutations in tet methylcytosine dioxygenase 2 (*TET2*) are another example that may result in increased self-renewal as they were described in clonal hematopoiesis without hematological malignancies [54]. Clonal expansion then favors additional mutations over time, eventually leading to secondary AML [53], [55]. Even in MDS, a single mutation scenario is unlikely and probably requires acquisition of additional growth advantage properties. For simplicity, our modeling does not reflect such clonal hierarchy evoked by multiple mutations. However, our models support the notion that the initial hit should result in higher cell intrinsic self-renewal rates.

Regulation of self-renewal appears to be more complex, and under physiologic conditions this feedback has to be lineage specific. In our model, we have implemented a single signal which relates to the number of stem and progenitor cells in the bone marrow niche. If the niche size was further limited to fewer compartments, then this would result in qualitatively similar results. In fact, the bone marrow cellularity in MDS is often increased [56] and this is also predicted by our models. The feedback signal might either be mediated systemically or locally by the microenvironment. In our previous work, we have demonstrated that serum obtained during aplasia after HSCT supported maintenance of a primitive immunophenotype in cultured cells (CD34^+^, CD133^+^, CD45^−^), and increased CFU-frequency as well as the number of cobblestone area-forming cells (CAFC) [31]. This led us to the assumption that self-renewal might be governed by serum factors, too. In this study, we demonstrate that addition of MDS serum results in faster decay of the primitive surface marker CD133 which is in agreement with assumptions of our model. We did not observe a clear effect on CFU-frequency, but definite demonstration of the effects of circulating factors on HSC self-renewal would require either serial colony replating assays or xenotransplantation of hematopoietic stem and progenitor cells following exposure to MDS serum. It is conceivable that the feedback signal is not serum-derived but directly mediated by the microenvironment. Several recent studies demonstrated that MDS cells closely interact with their niche and that MDS-derived MSCs might even reveal aberrations [13], [14]. With regard to the important interaction with niche cells, we have also considered co-culture experiments with MSCs. Stromal support greatly increased proliferation, but it did not display any effect related to MDS serum [34], [57]. It is conceivable that self-renewal is governed by a complex interplay of several molecules.

The nature of the signaling molecules, particularly for self-renewal, is still unclear. It is documented that hematopoietic stress rises concentrations of granulocyte-colony stimulating factor (G-CSF) and interleukin-6 (IL-6) in serum [58]. In MDS serum, elevated levels of adiponectin and osteocalcin and reduced levels of leptin, insulin and IGF-1 have been described [59]. Furthermore, EPO levels increase particularly in anemic patients [60] and this may contribute to the increased stimulation of erythroid colonies as observed in our study. However, it is also conceivable that other mediators, such as metabolites or exosomes, contribute to this regulation [61], [62]. A better understanding of the physiological regulatory processes will provide new perspectives for understanding the pathogenesis of MDS.

### Conclusion

Our study indicates that increased self-renewal of MDS-initiating cells is the most critical parameter to initiate MDS development. This may also explain why the disease seems to be stem cell derived as stem cells already reveal relatively high self-renewal rates. The central question in this process is the nature of feedback signals regulating hematopoiesis. Our models suggest that cure of MDS would only be achieved if the self-renewal rate can be specifically down-regulated in the malignant cells - particularly in the tumor-initiating MDS-LT-HSCs. Therefore, better understanding of the MDS-niche interaction is crucial to identify new therapeutic targets.

## Supporting Information

Text S1 Combined PDF file with Supplemental Methods, Supplemental Figures 1–6, and Supplemental Tables 1–2.
(PDF)
