# Supplementary material for: Feedback Signals in Myelodysplastic Syndromes: Increased Self-Renewal of the Malignant Clone Suppresses Normal Hematopoiesis
Source: PLoS Comput Biol. 2014 Apr 24;10(4):e1003599. doi: 10.1371/journal.pcbi.1003599 (PMC3998886; doi:10.1371/journal.pcbi.1003599)
Supplement: Text S1 — Combined PDF file with Supplemental Methods, Supplemental Figures 1–6, and Supplemental Tables 1–2. (PDF) [file pcbi.1003599.s001.pdf]

Text S1

Feedback Signals in Myelodysplastic Syndromes: Increased Self-Renewal of the Malignant Clone Suppresses Normal Hematopoiesis

Thomas Walenda [1,*], Thomas Stiehl [2,*], Hanna Braun [1], Julia Fröbel [3], Anthony D. Ho [4], Thomas Schroeder [3], Tamme W. Goecke [5], Björn Rath [6], Ulrich Germing [3], Anna Marciniak-Czochra [2] and Wolfgang Wagner [1]

1) Helmholtz Institute for Biomedical Engineering, RWTH Aachen University Medical School, Aachen, Germany;
2) Interdisciplinary Center of Scientific Computing (IWR), Institute of Applied Mathematics, University of Heidelberg, Heidelberg, Germany;
3) Department of Hematology, Oncology and Clinical Immunology, Heinrich-Heine-University, Düsseldorf, Germany;
4) Department of Medicine V, University Hospital Heidelberg, Germany;
5) Department of Obstetrics and Gynecology, RWTH Aachen University Medical School, Aachen, Germany;
6) Department for Orthopedics, RWTH Aachen University Medical School, Germany
*) equal contribution

Index

Supplemental Methods ... 2
  Mathematical modeling of normal hematopoiesis and MDS ... 2
  Derivation of the mathematical model ... 3
  Model of hematopoiesis in presence of dysplastic cells ... 5
  Derivation of the feedback signal in presence of dysplastic cells ... 6
  Immunophenotypic analysis ... 7
Supplemental Figures ... 8
  Figure 1: Simulation of MDS-development with alternative parameters. ... 9
  Figure 2: Detection of $CD34^+$ cells in MDS-bone marrow and normal controls ... 9
  Figure 3: Fold expansion of CB-derived $CD34^+$ cells. ... 9
  Figure 4: Immunophenotypic analysis upon co-culture with MSCs. ... 10
  Figure 5: MDS serum stimulates proliferation of BM-derived $CD34^+$ cells. ... 11
  Figure 6: Analysis of cytokine concentration in serum samples. ... 11
Supplemental Tables ... 12
  Table 1: Clinical data for serum samples ... 12
  Table 2: Patient data for bone marrow samples used in CD34 FACS analysis. ... 14
Supplemental References ... 15

Supplemental Methods

Mathematical modeling of normal hematopoiesis and MDS

The mathematical model developed in this study describes the interaction of physiological hematopoiesis and MDS cells. It is based on a previously proposed model of the hematopoietic system [1] that was extended to describe leukemia dynamics [2]. According to the classical understanding, hematopoiesis is assumed to be a multi-step process [3,4] in which cells move through an ordered sequence of different maturation stages (compartments). The following maturation stages are considered: (1) long-term repopulating stem cells (LT-HSCs), (2) short-term repopulating stem cells (ST-HSCs), (3) multi-potent progenitor cells (MPPs), (4) committed progenitor cells (CPCs), (5) precursors and (6) mature cells. For simplicity, we take only one hematopoietic lineage into account. We assume that daughter cells arising from division can either belong to the same type as the mother cell (self-renewal) or to a more mature cell type (differentiation). Time dynamics of each compartment are governed by cell gain due to self-renewal and by cell loss due to differentiation or apoptosis. The following cell properties are considered [1]: (1) proliferation rate, describing how many cell divisions take place per unit of time. We assume that mature blood cells and the most differentiated dysplastic cells are post-mitotic. (2) Fraction of self-renewal, describing which fraction of daughter cells belongs to the same maturation stage as their mother cell. (3) Apoptosis rate, describing which fraction of a cell population dies per unit of time.

It is further assumed that physiological hematopoiesis is regulated by feedback signals. In contrast to previous models, we consider two different feedback signals:
(1) The signal for proliferation is inversely correlated with the number of mature cells - resulting in higher proliferation upon cytopenia. This signal is motivated by the regulation of hematopoiesis by cytokines such as G-CSF or EPO, which increase if there is a shortage of mature cells [5,6]. For simplicity, we assume that this signal acts on all mitotic cells although some cell lineages and maturation stages are probably more sensitive than others. It is well known that many cytokines act on more than one cell type, e.g. G-CSF acts on the myeloid lineage and on HSC at the same time [6].
(2) The signal regulating self-renewal is dependent on the number of stem and progenitor cells in the marrow niche and acts on the cells residing in the niche. This signal is motivated by the fact that primitive cells require an appropriate microenvironment to maintain stemness [4,7]. We assume that if cells are outcompeted from this microenvironment, they can no longer perform self-renewal. For this reason, self-renewal rates decrease if the number of cells in the niche increases. For simplicity, we assume that the three most primitive cell types of each lineage are located in the bone marrow niche.

It is assumed that the MDS clone is maintained by cells with stem cell-like properties. It is known that MDS cells possess variable potential to differentiate into mature cells [8,9]. For simplicity, we assume in this model that no mature cells are produced by MDS cells (this does not account for the alternative model presented in Figure S1). Therefore, we consider five compartments of aberrant cells (MDS-LT-HSCs, MDS-ST-HSCs, MDS-MPPs, MDS-CPCs, and dysplastic progenitors). This maturation process is also regulated by the feedback signals mentioned above. We have tested various parameters for

the MDS clone. The results – and mathematical analysis [2] – demonstrate that the MDS-LT-HSC must reveal higher self-renewal potential than LT-HSCs for sustained disease progression. For different parameter values, qualitative behavior is very similar. Cell numbers and self-renewal rates for each compartment are chosen such that they adopt the same steady state behavior as described in our previous work [1]. For numerical simulations, we have used the ODE solver ode23t from Matlab (MathWorks, Natick, MA).

Derivation of the mathematical model

We use ordinary differential equations (ODEs) to describe interaction of hematopoietic and dysplastic cells. Time evolution of each maturation stage (compartment) is described by one ODE. We denote the concentration of healthy cells in maturation stage i at time t as $c_i(t)$, ($i=1,...,6$). For example, $c_1(t)$ denotes the number of LT-HSCs at time t, $c_6(t)$ the number of mature cells. Cell concentrations in the dysplastic cell line are denoted as $\hat{c}_j(t)$, $j=1,...,5$.

The proliferation rate of hematopoietic cells in stage i at time t is denoted as $p_i(t)$, that of dysplastic cells in stage j as $\hat{p}_j(t)$. Mature cells and dysplastic progenitors are considered to be post-mitotic: $p_6(t) \equiv 0$ and $\hat{p}_5(t) \equiv 0$ (this does not account for the alternative model in Figure 1 in Text S1). The fraction of self-renewal of hematopoietic cells in stage i at time t is denoted as $a_i(t)$ that of dysplastic cells in stage j as $\hat{a}_j(t)$. Analogously, the apoptosis rate of hematopoietic cells in stage i at time t is denoted as $d_i(t)$ and that of dysplastic cells in stage j as $\hat{d}_j(t)$. For simplicity, we assume that immature cells do not commit apoptosis, i.e., $d_i(t) \equiv 0$ for $i=1,...,5$ and that mature cells die at a constant positive rate $d_6(t) \equiv d_6 > 0$. In analogy, we set $\hat{d}_1(t) = ... = \hat{d}_4(t) \equiv 0$ for all t and $\hat{d}_5(t) \equiv \hat{d}_5 > 0$ (this does not account for the alternative model in Figure 1 in Text S1).

At time t, the flux to mitosis in hematopoietic compartment i ($i=1,...,5$) equals $p_i(t)c_i(t)$. This amount of cells disappears from compartment i and gives rise to $2p_i(t)c_i(t)$ daughter cells, $2a_i(t)p_i(t)c_i(t)$ of which stay in compartment i and $2(1-a_i(t))p_i(t)c_i(t)$ move on to compartment $i+1$.

We assume that proliferation and self-renewal change over time due to the feedback signals (s_1 and s_2). As derived from a quasi-steady-state approximation in our previous work [1], s_1 has the form $s_1(t) = \dfrac{1}{1+k \cdot c_6(t)}$. The positive constant k depends on the rates of cytokine production and

elimination. In analogy to our previous study [1], we assume that $p_i(t)$ (for $i = 1,...,5$) is given by $p_i(t) = p_{i,\max} \cdot s_1(t)$, where $p_{i,\max}$ is the maximal proliferation rate of cells in stage i. This regulation is a negative feedback: if there is a shortage of mature cells (i.e., $c_6(t)$ is small), proliferation rates increase and if mature cell counts are high, proliferation rates decrease.

We further assume that the number of primitive cells in BM niche regulates the fraction of self-renewal of the primitive cells. If it is assumed that self-renewal is regulated by the concentration $s_2(t)$ of signaling factors produced at constant rates and degraded by immature cells, we obtain $s_2(t) = \frac{1}{1 + l \cdot \sum_{i=1}^{3} c_i(t)}$ where l is a positive constant. In analogy to proliferation rates, we set $a_i(t) = a_{i,\max} \cdot s_2(t)$, for cells in the stages $i = 1,...,3$, where $a_{i,\max}$ is the maximal self-renewal fraction of cells in stage i. Self-renewal of more mature cell types is supposed to be constant and denoted by $a_i(t) \equiv a_i$, $i = 4,5$. Consequently, self- renewal increases if there is a shortage of primitive cells in the BM niche. Taking into account that mature cells are post-mitotic and that there exists no influx due to differentiation to the most primitive stage, we obtain for the hematopoietic lineage:

$$\frac{d}{dt} c_1(t) = \left(2a_{1,\max} s_2(t) - 1\right) p_{1,\max} s_1(t) c_1(t)$$

$$\frac{d}{dt} c_2(t) = 2\left(1 - a_{1,\max} s_2(t)\right) p_{1,\max} s_1(t) c_1(t) + \left(2a_{2,\max} s_2(t) - 1\right) p_{2,\max} s_1(t) c_2(t)$$

$$\frac{d}{dt} c_3(t) = 2\left(1 - a_{2,\max} s_2(t)\right) p_{2,\max} s_1(t) c_2(t) + \left(2a_{3,\max} s_2(t) - 1\right) p_{3,\max} s_1(t) c_3(t)$$

$$\frac{d}{dt} c_4(t) = 2\left(1 - a_{3,\max} s_2(t)\right) p_{3,\max} s_1(t) c_3(t) + \left(2a_4 - 1\right) p_{4,\max} s_1(t) c_4(t)$$

$$\frac{d}{dt} c_5(t) = 2\left(1 - a_4\right) p_{4,\max} s_1(t) c_4(t) + \left(2a_5 - 1\right) p_{5,\max} s_1(t) c_5(t)$$

$$\frac{d}{dt} c_6(t) = 2\left(1 - a_5\right) p_{5,\max} s_1(t) c_5(t) - d_6 c_6(t)$$

$$s_1(t) = \frac{1}{1 + k \cdot c_6(t)}$$

$$s_2(t) = \frac{1}{1 + l \cdot \sum_{i=1}^{3} c_i(t)}$$

The values of $k = 1.28 \cdot 10^{-9}$ and $l = 7.56 \cdot 10^{-9}$ were chosen such that the physiological equilibrium of mature cells is of the order of 10^9 cells / L of blood and such that proliferation rates could increase about 3 to 4 fold under regenerative pressure [10]. Parameters for the hematopoietic system were chosen as previously described [1,11].

Model of hematopoiesis in presence of dysplastic cells

We assume that, similar to the hematopoietic system, all dysplastic cells depended on the signal s_1 and that the three most primitive dysplastic cell types depend on the signal for self-renewal s_2. Including modulation of s_2 by primitive MDS-cells leads to the following expression for s_2:

$$s_2(t) = \frac{1}{1 + l \cdot \left(\sum_{i=1}^{3} c_i(t) + \sum_{j=1}^{3} \hat{c}_j(t) \right)}.$$

The positive constant l depends on production or elimination of signal molecules. This expression is motivated below.

For the interaction of healthy and dysplastic cells, the following ODE system is obtained:

$$\frac{d}{dt} c_1(t) = \left(2a_{1,\max} s_2(t) - 1\right) p_{1,\max} s_1(t) c_1(t)$$

$$\frac{d}{dt} c_2(t) = 2\left(1 - a_{1,\max} s_2(t)\right) p_{1,\max} s_1(t) c_1(t) + \left(2a_{2,\max} s_2(t) - 1\right) p_{2,\max} s_1(t) c_2(t)$$

$$\frac{d}{dt} c_3(t) = 2\left(1 - a_{2,\max} s_2(t)\right) p_{2,\max} s_1(t) c_2(t) + \left(2a_{3,\max} s_2(t) - 1\right) p_{3,\max} s_1(t) c_3(t)$$

$$\frac{d}{dt} c_4(t) = 2\left(1 - a_{3,\max} s_2(t)\right) p_{3,\max} s_1(t) c_3(t) + \left(2a_4 - 1\right) p_{4,\max} s_1(t) c_4(t)$$

$$\frac{d}{dt} c_5(t) = 2\left(1 - a_4\right) p_{4,\max} s_1(t) c_4(t) + \left(2a_5 - 1\right) p_{5,\max} s_1(t) c_5(t)$$

$$\frac{d}{dt} c_6(t) = 2\left(1 - a_5\right) p_{5,\max} s_1(t) c_5(t) - d_6 c_6(t)$$

$$\frac{d}{dt} \hat{c}_1(t) = \left(2\hat{a}_{1,\max} s_2(t) - 1\right) \hat{p}_{1,\max} s_1(t) \hat{c}_1(t)$$

$$\frac{d}{dt} \hat{c}_2(t) = 2\left(1 - \hat{a}_{1,\max} s_2(t)\right) \hat{p}_{1,\max} s_1(t) \hat{c}_1(t) + \left(2\hat{a}_{2,\max} s_2(t) - 1\right) \hat{p}_{2,\max} s_1(t) \hat{c}_2(t)$$

$$\frac{d}{dt} \hat{c}_3(t) = 2\left(1 - \hat{a}_{2,\max} s_2(t)\right) \hat{p}_{2,\max} s_1(t) \hat{c}_2(t) + \left(2\hat{a}_{3,\max} s_2(t) - 1\right) \hat{p}_{3,\max} s_1(t) \hat{c}_3(t)$$

$$\frac{d}{dt} \hat{c}_4(t) = 2\left(1 - \hat{a}_{3,\max} s_2(t)\right) \hat{p}_{3,\max} s_1(t) \hat{c}_3(t) + \left(2\hat{a}_4 - 1\right) \hat{p}_{4,\max} s_1(t) \hat{c}_4(t)$$

$$\frac{d}{dt}\hat{c}_5(t) = 2(1-\hat{a}_4)\hat{p}_{4,\max} s_1(t)\hat{c}_4(t) - \hat{d}_5\hat{c}_5(t)$$

$$s_1(t) = \frac{1}{1 + k \cdot c_6(t)}$$

$$s_2(t) = \frac{1}{1 + l \cdot \left(\sum_{i=1}^{3} c_i(t) + \sum_{j=1}^{3} \hat{c}_j(t) \right)}$$

By setting $\hat{c}_j(t) \equiv 0$, for $j = 1,...,3$, the system describing healthy hematopoiesis is obtained.

Derivation of the feedback signal in presence of dysplastic cells

The concentration of the signal regulating self-renewal at time t is denoted by $S(t)$. We assume that self-renewal is the default pathway with signal production at constant rate α_S. Degradation of this signal is assumed to be proportional to the cells in the bone marrow niche at a rate β_S. We further assume a cell independent degradation at rate γ_S. In analogy, we assume that dysplastic cells secrete an inhibitor I proportional to their concentrations at a rate α_I and that this inhibitor is degraded at a constant rate γ_I. The complex of inhibitor and signal molecules, denoted by IS, is supposed to be formed at a rate k_1. After formation of the complex, the signal molecule is degraded at a rate k_2. For simplicity, backward reactions are neglected. Dynamics of signal and inhibitor are described by the following ODE system:

$$\frac{d}{dt} I(t) = \alpha_I \sum_{j=1}^{3} \hat{c}_j(t) - \gamma_I \cdot I(t) - I(t) \cdot S(t) \cdot k_1 + IS(t) \cdot k_2$$

$$\frac{d}{dt} IS(t) = k_1 \cdot I(t) \cdot S(t) - k_2 \cdot IS(t)$$

$$\frac{d}{dt} S(t) = \alpha_S - \beta_S \left(\sum_{i=1}^{3} c_i(t) + \sum_{j=1}^{3} \hat{c}_j(t) \right) S(t) - \gamma_S S(t) - k_1 \cdot I(t) \cdot S(t)$$

We assume that molecular dynamics are fast in comparison to cell proliferation and differentiation, i.e. they are in a quasi-steady-state. We denote steady state concentrations of I, S and IS in the above system as $\bar{I}$, $\bar{S}$ and $\overline{IS}$. We express them as a function of the level of hematopoietic and dysplastic cells. It holds:

$$\bar{I} = \frac{\alpha_I \sum_{j=1}^{3} \hat{c}_j(t)}{\gamma_I}$$ and

$$\bar{S} = \frac{\alpha_S}{\beta_S\left(\sum_{i=1}^{3} c_i(t) + \sum_{j=1}^{3} \hat{c}_j(t)\right) + \gamma_S + k_1 \cdot \frac{\alpha_I \sum_{j=1}^{3} \hat{c}_j(t)}{\gamma_I}}.$$

Renormalization with respect to its maximal possible value yields

$$s(t) := \frac{\gamma_S}{\alpha_S}\bar{S} = \frac{1}{\frac{\beta_S}{\gamma_S}\left(\sum_{i=1}^{3} c_i(t) + \sum_{j=1}^{3} \hat{c}_j(t)\right) + 1 + \frac{k_1 \cdot \alpha_I}{\gamma_S \cdot \gamma_I} \cdot \sum_{j=1}^{3} \hat{c}_j(t)},$$

which is between zero and one. If we assume that production rates of the inhibitor are small, we obtain $s(t) \approx s_2(t)$ which is used in the model.

Immunophenotypic analysis

Cells were washed in PBS, stained with CD34-allophycocyanin (APC; Becton Dickinson, San Jose, CA, USA [BD], clone 8G12), CD133-phycoerythrin (PE, Miltenyi Biotec, Bergisch Gladbach, clone AC141) and CD45-V500 (BD, clone HI30) in a dilution of 1:200 and analyzed using a FACS Canto II (BD) running FACS Diva software (BD). Further analysis was performed using WinMDI software (WinMDI 2.8; The Scripps Institute, San Diego, CA, USA). Discrimination between MSC and HPC was possible by forward scatter, side scatter, propidium iodide (PI) staining and CFSE-staining. Immunophenotypic data of MDS-BM samples was obtained from 52 routinely screened MDS and 13 healthy control samples from the University Hospital Düsseldorf. The samples were stained with CD34-PE (BD, clone 8G12) and CD45-APC (BD, clone 2D1) in a dilution of 1:200 and analyzed using a FACS Calibur (BD) and FCS Express V3 software (De Novo Software, Los Angeles, CA, USA). The percentages of cells positive for CD34 were extracted and analyzed. Relevant patient data are shown in Table 2 in Text S1.

Supplemental Figures

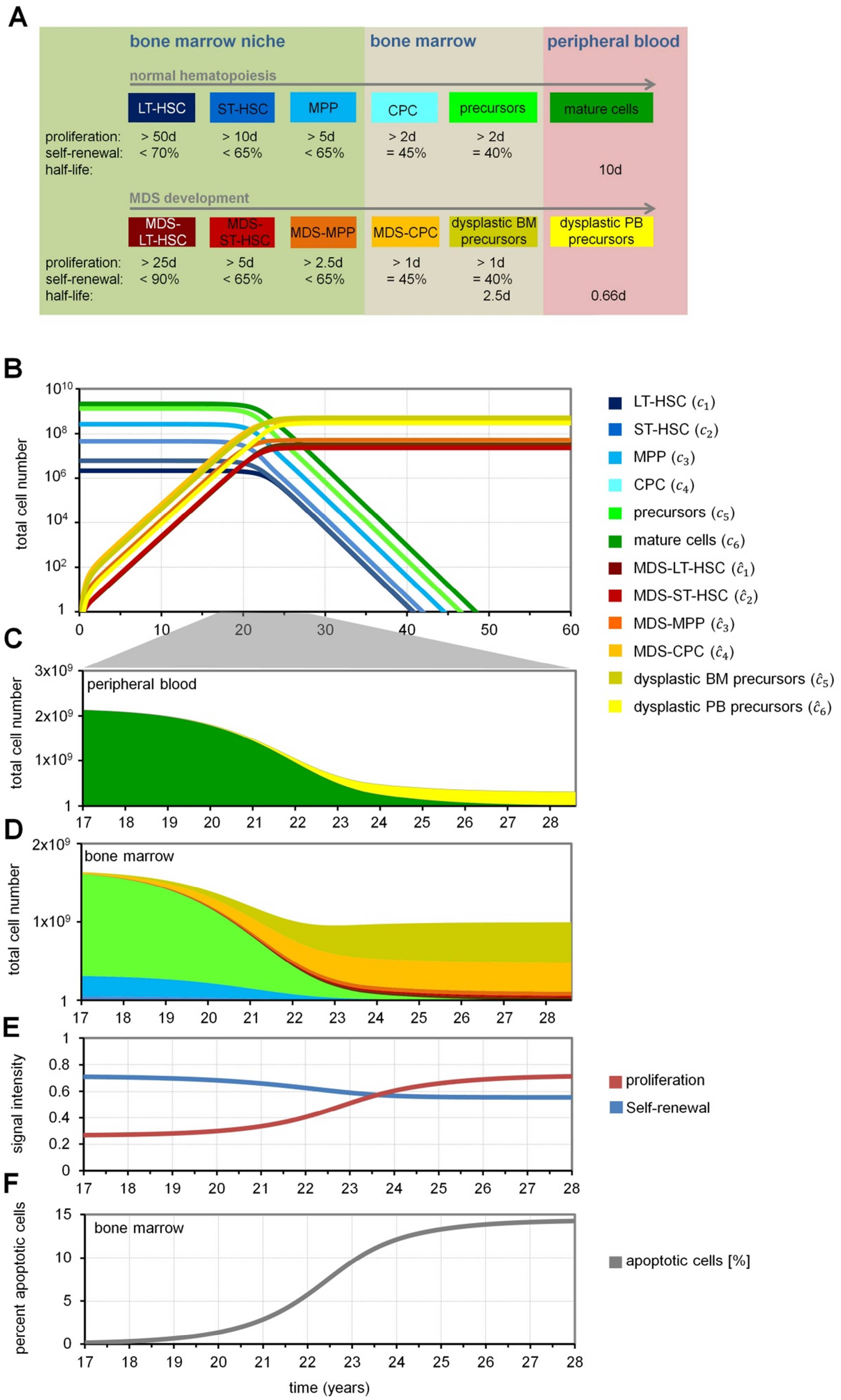

Legend on next page

Figure 1: Simulation of MDS-development with alternative parameters.

(A) In comparison to parameters of Figure 1, we used slightly different parameters for this alternative simulation: 1) MDS cells can progress to a dysplastic mature stage in peripheral blood which then also feeds back on proliferation, i.e. $S_1 = \frac{1}{(1+k(c_6+\hat{c}_6))}$; 2) proliferation rate of MDS cells of all differentiation stages is much higher than in normal hematopoiesis; and 3) dysplastic progenitors and dysplastic mature cells both undergo apoptosis at a much shorter half-life as compared to normal mature cells. This is in accordance with higher apoptosis rates observed in MDS patient bone marrow and peripheral blood [12,13,14]. The corresponding parameters are indicated for each cell type.

(B) Simulated MDS development over 60 years. (C) A sharp decline of mature cells is achieved after about 20 years and this would correspond to clinical manifestation of MDS. (D) Simulated cellular composition in the BM at the relevant time frame. Bone marrow becomes hypocellular due to high apoptosis rates of dysplastic cells. (E) Corresponding signal intensities for self-renewal and proliferation are presented. Self-renewal decays due to the accumulation of malignant cells in the BM-niche; proliferation is activated due to ineffective hematopoiesis. (F) The percentage of apoptotic cells in the bone marrow is presented. It increases in the course of simulated MDS development. For simulations, k and I were chosen as for the model presented above.

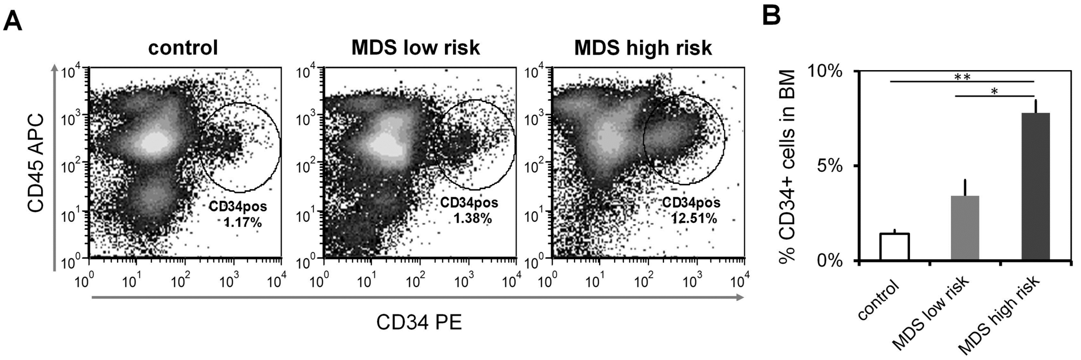

Figure 2: Detection of CD34$^+$ cells in MDS-bone marrow and normal controls.

(A) Example of presentation of CD34 measurement in healthy bone marrow, low-risk MDS and high risk MDS. The percentage of positive cells is depicted. (B) 52 MDS and 13 healthy BM-samples were analyzed with regard to CD34 and CD45 expression. High percentages of CD34$^+$ cells in BM samples correlate with bad disease prognosis. Relevant patient data are provided in Table 2 in Text S1. Error bars represent SEM (*p < 0.05, **p < 0.01).

Figure 3: Fold expansion of CB-derived CD34$^+$ cells.

Cells were expanded for 13 days in medium supplemented with SCF, TPO and FGF either without or with stromal support. Mean expansion rates were 60-fold and 242-fold, respectively.

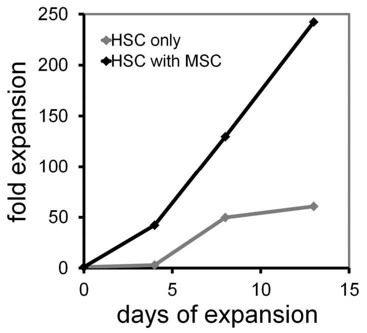

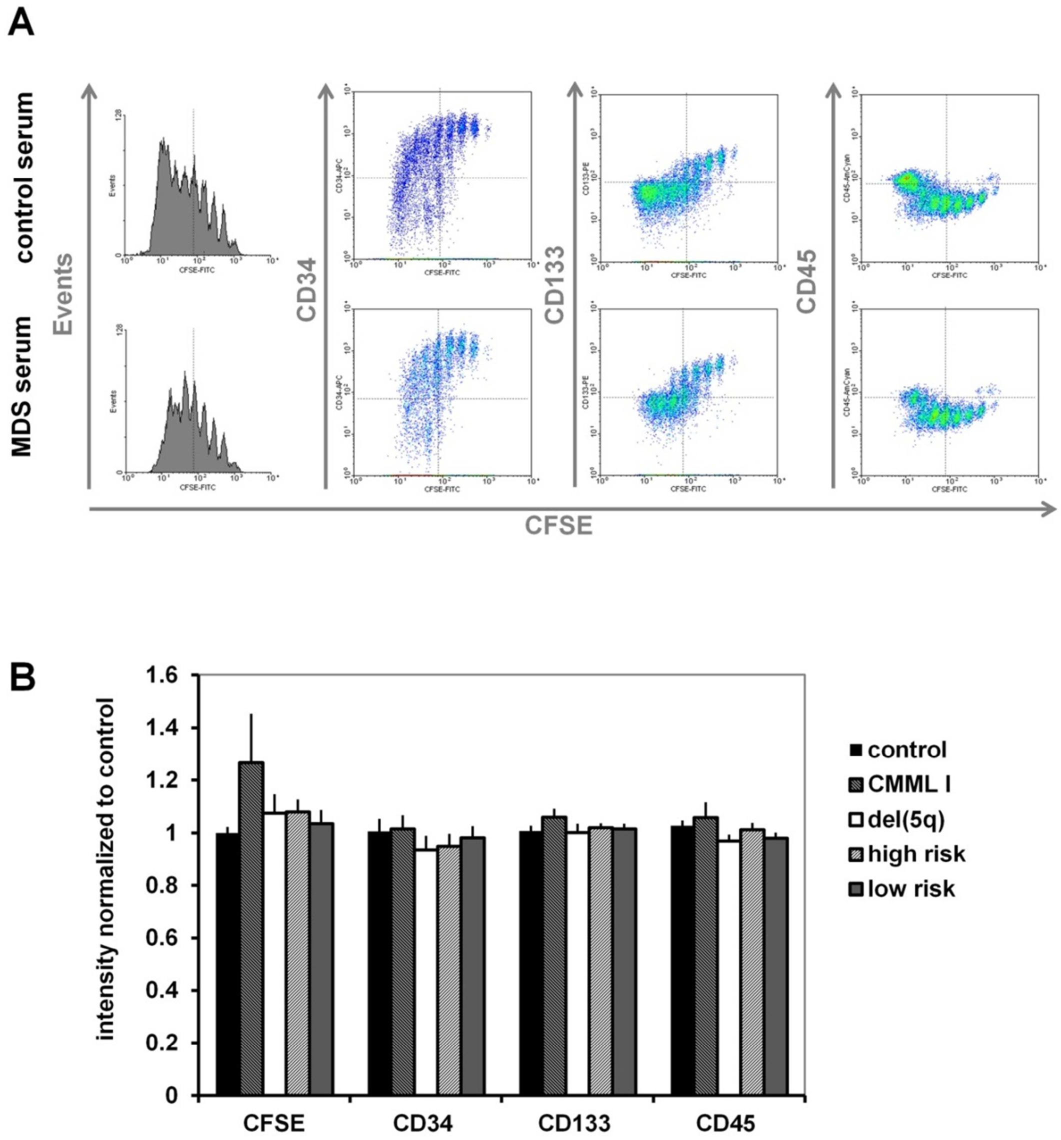

Figure 4: Immunophenotypic analysis upon co-culture with MSCs.

(A) $CD34^+$ CB-HPCs were stained with CFSE and subsequently cultivated for seven days *in vitro* on a confluent layer of MSCs. Culture medium was additionally supplemented with 10% serum from healthy controls (upper panel) or 10% serum from MDS-patients (lower panel). Flow cytometric analysis was used to monitor cell division history by residual CFSE-staining (dotted line in histogram indicates five cell divisions). Expression of CD34, CD45 and CD133 was analyzed in relation to the number of cell divisions. Representative histograms and density plots are demonstrated. (B) Analysis of mean fluorescence intensities after five cell divisions normalized to the control samples. No significant differences were detected. Error bars represent SD.

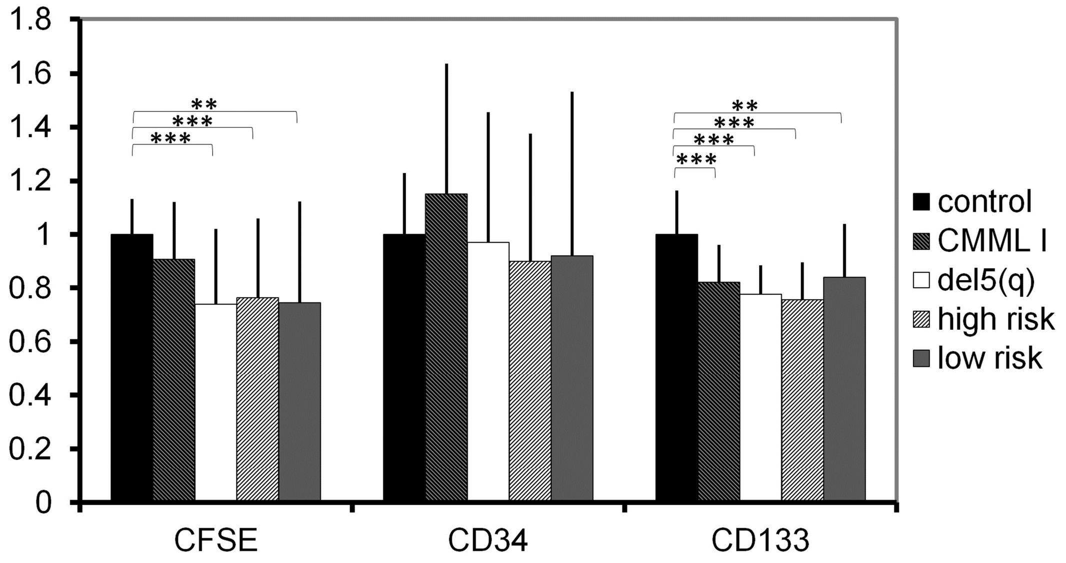

Figure 5: MDS serum stimulates proliferation of BM-derived CD34+ cells.

CD34+ HPCs from bone marrow were stained with CFSE and subsequently cultivated for four days *in vitro* in culture medium supplemented with 10% of individual patient or control serum. Mean fluorescence intensities after five cell divisions were normalized to control samples. Error bars represent SD (*p < 0.05, **p < 0.01, ***p < 0.001).

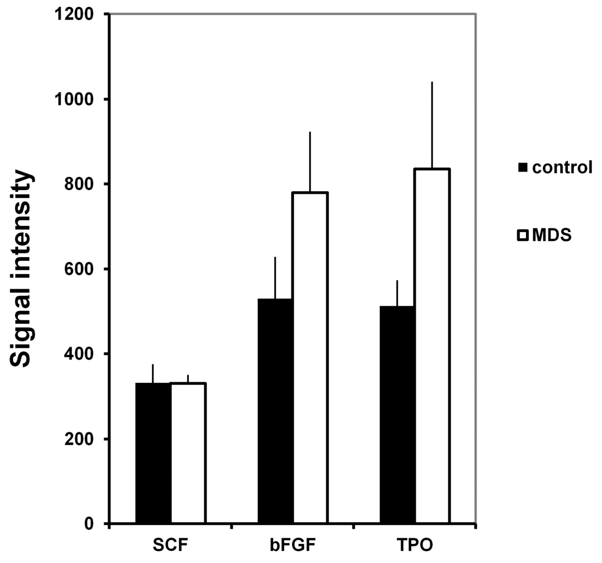

Figure 6: Analysis of cytokine concentration in serum samples.

Concentrations of SCF, bFGF and TPO in MDS-serum and healthy control serum samples were detected using RayBio Human ELISA Kits. Although there are tendencies to a higher amount of bFGF and TPO in MDS-serum samples, these differences were not statistically significant. Error bars represent SD.

Supplemental Tables

Table 1: Clinical data for serum samples.

ID	sex	age	MDS-subtype	leukocytes [/µL]	erythroc. [$1x10^6$/µL]	thromboc. [x1000/µL]	HB [g/dL]	BM-cellularity
1	m	51	RCMD	n.d.	n.d.	n.d.	n.d.	n.d.
2	m	73	RCMD	4000	n.d.	410	9.2	n.d.
3	f	66	RCMD	2500	4.98	83	12.4	n.d.
4	m	81	RCMD	5600	3.54	101	10.6	n.d.
5	m	75	RCMD	2400	2.31	87	9	n.d.
6	m	69	RAEB I	3100	2.53	51	10.5	normocellular
7	m	76	del(5q)	5500	4.00	165	13.2	hypocellular
8	m	74	RCMD	2800	2.25	391	6.7	normocellular
9	m	67	RCMD	6100	3.42	338	11.7	hypercellular
10	f	70	del(5q)	6000	4.77	117	14.4	normocellular
11	f	83	RCMD	3700	2.97	22	9.7	hypocellular
12	m	44	CMML I	5700	5.30	30	16	hypocellular
13	m	53	RAEB II	2500	3.69	40	10.7	hypocellular
14	m	73	sAML	1800	3.13	12	9.1	normocellular
15	f	85	del(5q)	9700	3.00	397	10	hypercellular
16	m	76	RAEB II	2700	3.28	80	10	hypercellular
17	f	72	del(5q)	2270	2.27	77	6.1	hypercellular
18	m	49	RCMD	9300	2.85	88	8	hypercellular
19	f	71	del(5q)	4700	2.86	55	10.4	normocellular
20	m	60	RCMD	3100	2.98	133	10.5	n.d.
21	m	71	RAEB I	2600	3.73	268	13.1	n.d.
22	m	57	RCMD	18100	2.43	73	7.3	hypercellular
23	f	61	RCMD	3900	2.69	262	8.4	normocellular
24	f	61	RCMD	16400	2.96	344	8.7	hypocellular
25	m	67	sAML	13600	2.99	12	8.8	hypercellular
26	f	92	RCMD-RS	6200	1.66	505	5.7	hypercellular
27	m	64	RCMD	10200	2.37	283	9.8	hypercellular
28	f	68	RAEB I	800	3.42	13	9.8	n.d.
29	f	71	CMML I	23800	4.66	115	13.8	normocellular
30	m	71	RAEB II	1700	3.88	103	10.8	n.d.
31	m	77	sAML	43700	2.77	31	7.7	n.d.
32	f	64	RCMD	3800	2.95	162	9.5	n.d.
33	m	70	CMML I	4600	4.06	57	12.1	normocellular
34	m	57	CMML I	4300	4.61	144	14.1	hypercellular
35	m	77	CMML I	5800	3.25	102	8.5	hypercellular
36	f	73	CMML I	3800	4.48	80	12.5	n.d.
37	f	59	RCMD	1900	1.85	387	7.2	hypercellular
38	f	83	RCMD-RS	2000	2.47	265	8.4	normocellular
39	m	74	RCMD	3000	3.79	18	10.6	hypercellular

40	m	77	CMML I	10300	5.76	192	15.4	hypercellular
41	f	65	CMML I	1800	3.54	74	10.7	n.d.
42	m	60	del(5q)	14600	2.31	734	7.3	n.d.
43	m	58	RAEB I	3200	4.68	231	13.9	hypercellular
44	m	62	RCMD	1900	2.42	236	6.9	hypercellular
45	f	54	RAEB I	800	3.19	22	10.4	n.d.
46	m	73	RCMD	2500	3.03	22	8.5	normocellular
47	m	76	RCMD	1200	2.97	36	9.1	normocellular
48	f	80	CMML I	184000	2.74	37	8.2	n.d.
49	f	75	RCMD	11000	2.19	420	7.9	hypercellular
50	m	72	CMML I	5600	3.69	62	13.7	hypercellular
51	f	68	RAEB I	2200	2.81	100	10.2	hypocellular
52	f	77	RAEB II	2600	3.09	90	9	hypercellular
53	m	74	RCMD-RS	2690	2.69	374	9.5	normocellular
54	m	68	RCMD-RS	4100	3.39	263	9.5	n.d.
55	f	65	sAML	1500	2.70	102	8.7	n.d.
56	m	76	RCMD	5800	3.06	10	9.9	hypercellular
57	m	81	RCMD	12800	3.35	364	9	hypercellular

Data from healthy controls

H1	m	46	healthy	7100	5.2	269	16.2	n.d.
H2	m	61	healthy	7400	4.17	180	13.9	n.d.
H3	m	71	healthy	8200	4.87	219	15.7	n.d.
H4	f	73	healthy	5000	3.92	378	11.9	n.d.
H5	m	61	healthy	7400	3.71	464	12.1	n.d.
H6	f	74	healthy	5600	4.7	199	14.3	n.d.
H7	f	68	healthy	4100	4.5	313	14.4	n.d.
H8	f	34	healthy	4600	5	250	14.8	n.d.
H9	f	40	healthy	9900	4.4	216	13.2	n.d.
H10	f	48	healthy	9400	2.9	335	8.2	n.d.
H11	f	51	healthy	n.d.	3.5	953	9.9	n.d.
H12	f	43	healthy	7400	5	279	14.8	n.d.

CMML (chronic myelomonocytic leukemia); del(5q) (chromosomal deletion 5q); RAEB (refractory anemia with excess of blasts); RCMD (refractory cytopenia with multilinear dysplasia); RCMD-RS (with ringed sideroblasts); sAML (secondary acute myeloid leukemia), n.d. (not determined); all patients were treated without involvement of erythropoietin.

Table 2: Patient data for bone marrow samples used in CD34 FACS analysis.

ID	sex	age	MDS-subtype	leukocytes [/µL]	erythroc. [1x10^6/µL]	thromboc. [x1000/µL]	HB [g/dL]	BM-cellularity
F1	m	76	RCMD	3600	2.06	355	8.5	n.d.
F2	m	69	RAEB II	3200	3.52	175	10.0	n.d.
F3	f	69	RCMD	4800	2.00	402	7.2	n.d.
F4	m	76	del(5q)	5500	4.00	165	13.2	hypocellular
F5	f	71	RAEB II	1800	3.03	17	7.6	n.d.
F6	m	70	RCMD	1400	2.84	51	8.1	n.d.
F7	m	53	RCMD	5300	3.06	192	9.0	n.d.
F8	m	62	CMML II	16700	3.39	87	10.2	n.d.
F9	f	67	RAEB II	1600	2.57	80	9.5	n.d.
F10	m	79	RCMD-RS	3000	3.23	215	9.5	n.d.
F11	f	49	RAEB II	6900	3.47	160	10.6	n.d.
F12	m	64	RAEB II	900	3.86	149	11.0	n.d.
F13	m	76	RAEB II	1000	3.38	64	8.9	n.d.
F14	f	65	RAEB II	2400	3.20	50	11.0	n.d.
F15	m	75	RAEB II	1300	2.78	23	10.4	n.d.
F16	m	73	sAML	1800	3.13	12	9.1	normocellular
F17	m	65	RCMD	1900	2.87	113	8.6	n.d.
F18	f	68	RCMD	1700	3.45	24	12.7	n.d.
F19	m	71	RCMD	3200	3.38	52	10.7	n.d.
F20	m	78	RCMD	3300	3.62	70	9.0	n.d.
F21	m	62	RCMD	1900	2.42	236	6.9	hypercellular
F22	f	72	RAEB II	6100	2.89	33	8.8	n.d.
F23	m	79	RCMD	2800	2.59	306	8.0	n.d.
F24	m	53	RAEB II	2500	3.69	40	10.7	hypocellular
F25	m	68	RCMD-RS	4100	3.39	263	9.5	n.d.
F26	f	65	CMML I	1800	3.54	74	10.7	n.d.
F27	f	76	CMML II	9400	2.80	198	9.8	n.d.
F28	m	64	RCMD	10200	2.37	283	9.8	hypercellular
F29	f	42	RARS	3300	2.75	468	9.8	n.d.
F30	m	49	RCMD	9300	2.85	88	8.0	hypercellular
F31	f	71	RCMD	4200	2.69	97	8.7	n.d.
F32	m	38	RAEB II	2500	2.96	50	9.3	n.d.
F33	m	51	RAEB II	2400	3.86	32	11.3	n.d.
F34	m	65	RCMD	6600	2.98	302	9.9	n.d.
F35	f	60	RCMD	2700	3.25	407	10.1	n.d.
F36	m	62	RAEB II	4100	3.11	30	8.8	n.d.
F37	m	59	RCMD	4800	3.06	159	11.4	n.d.
F38	m	69	RAEB I	3100	2.53	51	10.5	normocellular
F39	m	63	RARS	3700	2.50	248	7.4	n.d.
F40	m	60	del(5q)	14600	2.31	734	7.3	n.d.
F41	m	71	RAEB I	2000	2.88	86	9.2	n.d.
F42	f	73	RCMD	1600	3.02	20	9.9	n.d.
F43	m	70	RCUD	10400	4.71	148	13.6	n.d.
F44	f	68	RCMD	4900	3.27	158	10.0	n.d.
F45	m	74	RCMD	1100	2.56	33	9.8	n.d.

F46	m	47	CMML I	5000	3.65	69	10.5	n.d.
F47	f	66	RCMD	3800	2.31	119	7.1	n.d.
F48	m	69	RCMD	10800	3.01	13	8.8	n.d.
F49	m	70	RCMD	1500	3.41	47	10.3	n.d.
F50	m	72	CMML I	5600	3.69	62	13.7	hypercellular
F51	m	83	del(5q)	2600	3.48	81	10.1	n.d.
F52	m	75	RCMD	2800	2.25	391	6.7	normocellular
**Data from healthy controls:**								
HF1	f	41	healthy	6000	4.22	158	13.1	n.d.
HF2	m	55	healthy	5600	3.51	90	10.6	n.d.
HF3	n.d.	22	healthy	11200	3.73	438	10.5	n.d.
HF4	f	82	healthy	n.d.	n.d.	n.d.	n.d.	n.d.
HF5	f	59	healthy	3200	2.94	35	9.7	n.d.
HF6	m	22	healthy	9600	3.91	165	13.1	n.d.
HF7	f	36	healthy	7000	3.24	234	9.4	n.d.
HF8	m	71	healthy	11800	3.28	44	9	n.d.
HF9	m	75	healthy	n.d.	n.d.	n.d.	n.d.	n.d.
HF10	m	71	healthy	4000	2.97	88	8.6	n.d.
HF11	m	81	healthy	4200	3.18	47	9.9	n.d.
HF12	f	53	healthy	3300	4.73	143	12.4	n.d.
HF13	f	63	healthy	2100	3.76	54	9.9	n.d.

CMML (chronic myelomonocytic leukemia); del(5q) (chromosomal deletion 5q); RAEB (refractory anemia with excess of blasts); RCMD (refractory cytopenia with multilinear dysplasia); RCMD-RS (with ringed sideroblasts); sAML (secondary acute myeloid leukemia), n.d. (not determined).

Supplemental References

1. Marciniak-Czochra A, Stiehl T, Ho AD, Jaeger W, Wagner W (2009) Modeling of Asymmetric Cell Division in Hematopoietic Stem Cells - Regulation of Self-Renewal is Essential for Efficient Repopulation. Stem Cells Dev 18: 377-385.

2. Stiehl T, Marciniak-Czochra A (2012) Mathematical modeling of leukemogenesis and cancer stem cell dynamics. Mathematical Modelling of Natural Phenomena 7: 166-202.

3. Larsson J, Karlsson S (2005) The role of Smad signaling in hematopoiesis. Oncogene 24: 5676-5692.

4. Ho AD, Wagner W (2007) The beauty of asymmetry - asymmetric divisions and self-renewal in the hematopoietic system. Current Opinion in Hematology 14: 330-336.

5. Layton JE, Hockman H, Sheridan WP, Morstyn G (1989) Evidence for a novel in vivo control mechanism of granulopoiesis: mature cell-related control of a regulatory growth factor. Blood 74: 1303-1307.

6. Metcalf D (2008) Hematopoietic cytokines. Blood 111: 485-491.

7. Warr MR, Pietras EM, Passegue E (2011) Mechanisms controlling hematopoietic stem cell functions during normal hematopoiesis and hematological malignancies. Wiley Interdiscip Rev Syst Biol Med 3: 681-701.

8. Will B, Zhou L, Vogler TO, Ben-Neriah S, Schinke C, et al. (2012) Stem and progenitor cells in myelodysplastic syndromes show aberrant stage-specific expansion and harbor genetic and epigenetic alterations. Blood 120: 2076-2086.

9. Agarwal A (2012) MDS: roadblock to differentiation. Blood 120: 1968-1969.

10. Thornley I, Sutherland DR, Nayar R, Sung L, Freedman MH et al. (2001) Replicative stress after allogeneic bone marrow transplantation: changes in cycling of CD34+CD90+ and CD34+. Blood 97: 1876-1878.

11. Marciniak-Czochra A, Stiehl T, Wagner W (2009) Modeling of replicative senescence in hematopoietic development. Aging 1: 723-732.

12. Pang WW, Pluvinage JV, Price EA, Sridhar K, Arber DA et al. (2013) Hematopoietic stem cell and progenitor cell mechanisms in myelodysplastic syndromes. Proc Natl Acad Sci USA 110: 3011-3016.

13. Pecci A, Travaglino E, Klersy C, Invernizzi R (2003) Apoptosis in relation to CD34 antigen expression in normal and myelodysplastic bone marrow. Acta Haematol 109: 29-34.

14. Houwerzijl EJ, Blom NR, van der Want JJ, Louwes H, Esselink MT et al. (2005) Increased peripheral platelet destruction and caspase-3-independent programmed cell death of bone marrow megakaryocytes in myelodysplastic patients. Blood 105: 3472-3479.
